# Genome sequencing reveals coinfection by multiple chikungunya virus genotypes in a recent outbreak in Brazil

**DOI:** 10.1371/journal.pntd.0007332

**Published:** 2019-05-16

**Authors:** Lais Ceschini Machado, Mariana Carolina de Morais-Sobral, Tulio de Lima Campos, Mylena Ribeiro Pereira, Maria de Fátima Pessoa Militão de Albuquerque, Clément Gilbert, Rafael Freitas Oliveira Franca, Gabriel Luz Wallau

**Affiliations:** 1 Department of Entomology, Oswaldo Cruz Foundation - Fiocruz, Aggeu Magalhães Institute - Recife, Pernambuco - Brazil; 2 Bioinformatics Core Facility, Aggeu Magalhães Institute (IAM), Oswaldo Cruz Foundation (FIOCRUZ), Recife Pernambuco, Brazil; 3 Department of Veterinary Medicine, Federal Rural University of Pernambuco – UFRPE, Recife, Brazil; 4 Department of Public Health, Oswaldo Cruz Foundation - Fiocruz, Aggeu Magalhães Institute - Recife, Pernambuco - Brazil; 5 Laboratoire Evolution, Génomes, Comportement, Ecologie, CNRS Université Paris-Sud UMR 9191, IRD UMR 247, Avenue de la Terrasse, Gif sur Yvette, France; 6 Department of Virology and Experimental Therapy, Oswaldo Cruz Foundation - Fiocruz, Aggeu Magalhães Institute - Recife, Pernambuco - Brazil; Hôpital Laveran, FRANCE

## Abstract

Chikungunya virus (CHIKV) is an RNA virus from the Togaviridae family transmitted by mosquitoes in both sylvatic and urban cycles. In humans, CHIKV infection leads to a febrile illness, denominated Chikungunya fever (CHIKF), commonly associated with more intense and debilitating outcomes. CHIKV arrived in Brazil in 2014 through two independent introductions: the Asian/Caribbean genotype entered through the North region and the African ECSA genotype was imported through the Northeast region. Following their initial introduction, both genotypes established their urban cycle among large naive human populations causing several outbreaks in the Americas. Here, we sequenced CHIKV genomes from a recent outbreak in the Northeast region of Brazil, employing an in-house developed Next-Generation Sequencing (NGS) protocol capable of directly detecting multiple known CHIKV genotypes from clinical positive samples. Our results demonstrate that both Asian/Caribbean and ECSA genotypes expanded their ranges, reaching cocirculation in the Northeast region of Brazil. In addition, our NGS data supports the findings of simultaneous infection by these two genotypes, suggesting that coinfection might be more common than previously thought in highly endemic areas. Future efforts to understand CHIKV epidemiology should thus take into consideration the possibility of coinfection by different genotypes in the human population.

## Author summary

Human infection by different arthropod borne viruses such as Chikungunya, Dengue and Zika causes a continuous and extensive health burden mainly in tropical regions of the world. Most of the CHIKV infections result in only mild symptoms, however more extreme manifestations can occur, occasionally leading to death. Different CHIKV genotypes have been circulating in almost all continent around the globe but so far human outbreaks were reported only for single CHIKV genotypes. In Brazil two CHIKV genotypes arrived in the North and Northeast region: the Asian/Caribbean and African ECSA respectively. Here, through viral genome sequencing, we showed that some samples were coinfected by both Asian/Caribbean and ECSA CHIKV genotypes, indicating that these two genotypes expanded their ranges and currently co-circulate in some areas of Brazil. In summary, such findings call for improved surveillance in order to detect co-circulation of different viral genotypes and further evaluate the impact of co-infection in disease epidemiology.

## Introduction

Chikungunya virus (CHIKV) is a medically important arthropod-borne virus (arbovirus) from the Alphavirus genus and Togaviridae family, which is transmitted to humans through *Aedes aegypti* and *Ae*. *albopictus* bites [1,2]. This virus has a single stranded positive RNA genome with approximately 11.8-kb in length encoding two polyproteins responsible for replication and propagation [3]. CHIKV infection most commonly reported symptoms include fever and joint pain, which can last for a few months to several years, and a number of other manifestations such as bullous dermatitis and myocarditis. In addition, CHIKV infection can also cause neurological manifestations such as meningitis, myelitis, encephalitis and Guillain-Barré syndrome [4–8], although such outcomes are rare and mostly observed in patients showing pre-existing underlying diseases [7,9,10].

CHIKV was first isolated from an outbreak in Tanzania in 1952 [11] and phylogenomic analyses, with past and current lineages, revealed three distinct genotypes: West African (WA), East/Central/South African (ECSA) and Asian/Caribbean [12]. Both WA and ECSA genotypes were mainly maintained in enzootic cycles in the African continent, being transmitted among non-human primates, with sporadic spillover to humans, although some ECSA lineages expanded their range and are currently causing large outbreaks in several human populations living in tropical regions. The Asian/Caribbean genotype is mainly endemic/epidemic among human populations. Several CHIKV outbreaks have occurred across the globe due to the arrival of once geographically restricted genotypes into new areas with large naive human populations or in areas with previous circulation of other genotypes [13]. Three major outbreaks are well documented: I—An ECSA outbreak in Kenya in 2004, and further spreading to the Indian Ocean Islands, giving rise to the Indian Ocean Lineage (IOL); II—Several outbreaks of the Asian genotype in Pacific Islands followed by spreading to the Caribbean islands and mainland North and South America [14]; III—A recent emergence of ECSA genotype in the Northeast of Brazil through the arrival of a traveler from Angola in Feira de Santana city—Bahia state [15,16] and further spreading south and northwards [17–20]. Interestingly, the ECSA genotype lineage that entered Brazil was recently detected into both *Culex quinquefasciatus* and *Ae*. *albopictus* pools in the Caribbean island of Haiti [21] which was first hit by the Asian/Caribbean genotype.

Based on vector species habitat suitability, it was suggested that both Asian/Caribbean and ECSA genotypes would spread and co-circulate in countrywide Brazil [15]. So far, a single patient infected with the Asian/Caribbean genotype was detected in 2014 at the Pernambuco state, Northeast Brazil [15], however no other studies further confirmed the circulation of this genotype in this region. ECSA genotype was found in this same geographical location (personal communication—Gabriel Luz Wallau and GenBank KX228391 accession number) as well as in other states from the Northeast region, suggesting that both genotypes are simultaneously circulating in this area [17–19]. Albeit two hundred thousand CHIKV infection cases were reported in Brazil in 2016 the proportion and distribution of infected patients by each genotype as well as the possibility of coinfection is completely unknown. A recent study identified 132 CHIKV cases out of 263 samples initially screened for dengue (DENV) and Zika (ZIKV) viruses, showing that CHIKV was able to displace a previous ZIKV outbreak in Northeast Brazil between May 2015 to May 2016, but distinction of CHIKV genotypes were not determined [19].

In the present study, we sequenced the CHIKV genome directly from clinical samples and showed for the first time the presence of two CHIKV genotypes simultaneously circulating in the same region. Moreover, our results show evidence of coinfection of ECSA and Asian/Caribbean CHIKV genotypes in naturally infected human individuals. Lastly, molecular clock analysis estimated that the ECSA CHIKV genotypes spread Northwards around mid 2015, concomitantly to its initial detection in the Northeast region of Brazil.

## Methods

### Study participants and sampling procedures

Patients were recruited through a previous established study protocol seeking neurological disorders with history of arbovirus infection (personal communication—Rafael Freitas Oliveira Franca). Briefly, patients were recruited from a public hospital, which is in charge of 70% of neurological cases in the state of Pernambuco, Northeast Brazil. As inclusion criteria, patients presenting any neurological disease who reported a previous episode of rash had a blood sample collected. Patients with classical CHIKV symptoms, without neurological manifestation, presenting with fever, myalgia and joint pain were also included. Samples were further processed for laboratory diagnosis of recent arboviral infection by rRT-PCR (Real Time RT-PCR), employing established protocols for detection of DENV, ZIKV and CHIKV viruses. A detailed history and clinical examination, including a full neurological examination were conducted in these patients and complementary clinical exams were performed when necessary (brain imaging, lumbar puncture, nerve conduction studies and electromyography). Based on neurological evaluation, patients were grouped on nervous system disease manifestation as follows: Acute Transverse Myelitis—acute signs of neurologic dysfunction in motor, sensory, and autonomic nerves and nerve tracts of the spinal cord, Optic Neuritis—acute severe visual disturbance without any clear diagnostic findings on ocular examination and Polymyositis—severe muscular weakness. Written informed consent was provided by all patients enrolled in this study. All human subjects were adults. The study protocol was reviewed and approved by the Oswaldo Cruz Foundation—FIOCRUZ, Aggeu Magalhães Institute Ethics Committee (CAAE #511.06115.8 000 5190).

### Virus detection—Real Time RT-PCR

Viral RNA was extracted from serum samples by the use of QIAamp Viral RNA kit (Qiagen, Hilden—Germany) following manufacturer’s instructions. Eluted RNAs were stored at -80°C until use. Real Time RT-PCR (rRT-PCR) reactions were performed from purified RNA serum samples accordingly to Lanciotti et al., 2006 and 2008 [22–24].

### Viral genome sequencing

Total RNA was used for single strand cDNA generation employing Random Hexamers (Invitrogen) and ProtoScript II Reverse Transcriptase (New England Biolabs), following manufacturer instructions. cDNA were submitted to multiplex PCR with primers designed using Primal Scheme from the ZiBRA project (http://primal.zibraproject.org/) based on a multiple genome alignment of CHIKV sequences both from Asian/Caribbean and ECSA CHIKV strains detected in South America (GenBank accession numbers KP164576, KP164571, KP164572, KP164568, KP164570 and KP164569) available at the time. Primer sequences can be found in S1 Table. PCR were performed independently for 4 pools with Q5 Hot Start High-Fidelity DNA Polymerase (New England BioLabs). Cycle conditions were as follows: 98°C at 30 seconds, 98°C at 15 seconds, 61°C at 5 minutes and 65°C at 5 minutes for 45 cycles. PCR products were quantified using Qubit dsDNA HS Assay Kit (Thermo Fisher Scientific Inc.). Sequencing libraries were prepared with Nextera XT Library Prep Kit (Illumina, San Diego, CA, USA) using 2 ng of cDNA following manufacturer′s instructions. MiSeq Reagent Kit V3 of 150 cycles was used employing a paired-end strategy. Sequencing was performed in the MiSeq (Illumina) machine. Raw reads are available at the European Nucleotide Archive (ENA) under the project number PRJEB23381.

### Reads trimming, genome mapping and variant call

The raw “fastq” files were trimmed using Trimmomatic 0.36 [25] with the parameter SLIDINGWINDOW:15 and the Illumina MiSeq adapter and custom primers sequences used for fragments amplification were provided to remove those sequences from the reads. Due to the high variability found in our samples we were not able to use softwares for recombination detection such as RPD [26] and Simplot [27] since they are based on the consensus sequences of viral genomes. Such consensus sequences in our case would be very disturbed due to the variability found. Moreover, most methods developed to characterize coinfection/mutation cloud do not output a graphical view to interpret the variability position along the genome. Therefore, based on the reasoning of Simplot [27] and Deep Simplot [28], we developed an *in house* shell and R script (available in S1 Data) to extract the “NM” column from alignment “.bam” files generated by Bowtie2—default parameters [29], which reports the number of SNPs of each read mapped along with the position of the read in the reference genome. Both KP164568 from ECSA genotype and KP164571.1 from Asian/Caribbean genotype genomes were used to map the reads of every sample using Bowtie2. It is important to highlight that these ECSA and Asian/Caribbean CHIKV genomes detected circulating in South America are 90% identical at the nucleotide level but several genotype specific SNPs also could be detected between them which allow the genotyping of the reads mapped at the variable regions (S1 Fig shows the conserved and variable regions among the 3 ECSA and 3 Asian Caribbean genomes already detected in South America). Finally, ggplot2 [30] was used to plot the average distance of the reads to both genomes using a 100 bp sliding window which then allows the visualization of recombination and coinfection/quasispecies signals in the same graph. Finally, SNP calls were performed with samtools mpileup and vcf-annotate tools [31] with the following parameters (vcf-annotate—filter Qual = 20/MinDP = 200/SnpGap = 20) and snpEff software was used for SNP annotation. [32]

### Reads simulation

Read simulation was performed with ART [33] with the following parameters (art_illumina -ss MSv1 -p -l 75 -f 500 -m 200 -s 10 -qL 27), that is, generating 75bp paired-end reads, 500x of coverage depth, 200bp of median size DNA fragments for paired-end simulations, standard deviation of DNA fragment size of 10 and the minimum base quality score of 27. The reference genomes used for read simulations were the same ECSA (KP164568) and Asian/Caribbean (KP164571.1) genomes. In addition, we simulated two recombination events between these genomes: I—an ECSA 5’ region (7555bp) /Asian/Caribbean 3’ region (4646bp) genome; II—an Asian/Caribbean 5’ region (7555bp) /ECSA 3’ region (4257bp) genome; and a co-infection/quasispecies pattern all along the genome mixing equal proportion of reads generated from ECSA and Asian/Caribbean reference genomes reported above.

### Phylogenetic analysis

Phylogenetic tree were reconstructed using a bayesian inference approach with BEAST 1.8.0 [34] employing the GTR+I+G model indicated as the most likely model to represent the data by jModelTest 2.1.10 [35] with a relaxed lognormal molecular clock model and the Skyline tree prior. Consensus genomes from the samples with coinfection were generated with Integrated Genome Viewer (IGV) and all Asian/Caribbean SNPs were removed since the ECSA genotype genomes presented a larger genome coverage breadth. Six independent runs were performed with 50 million generations including the three genotypes (WAfr, Asian/Caribbean and ECSA) and 30 millions generations for ECSA focusing only on samples from South and Central America. Tree sampling was performed at every 1000 generations. Parameters convergence was evaluated by the effective sample size (ESS>200) using TRACER version 1.6 (Rambaut et al 2014). Tree visualization and figure generation was performed with Figtree 1.4.3.

## Results

### Draft genomes

We obtained CHIKV draft genomes from all eight samples screened presenting coverage breadth varying from 79.46% to 98.64% and an average depth from 2201 to 10538x (Table 1). Due to the fact that amplicon sequencing is prone to cross contamination we also sequenced two negative controls following the approach used by Metsky et al 2017 for ZIKV genome sequencing [36]. We detected some degree of contamination in both negative control samples varying from 1033x in Serum negative control sample to 1641x in H20 negative control of coverage depth (Table 1). These low levels of contamination are in the range detected by Metsky et al (2017) using this same amplicon-based protocol for Zika virus [36]. Negative controls showed a much lower genome coverage breadth than all positive CHIKV samples analyzed, in agreement with previous studies. Moreover, we could not identify in either control samples a similar variability pattern (reads from the Asian/Caribbean genotype—see **Polymorphism and coinfection** subsection below) detected in some positive CHIKV positive samples (.bam mapping output files can be found at figshare repository—https://figshare.com/s/b9e3d8848d4729ff3169). Therefore, it is unlikely that such low level of contamination has any impact on our results. Using a cutoff similar to Metsky et al (2017) we followed the analysis with all eight positive samples.

**Table 1:** Sequencing data generated for the eight samples and negative controls.

Sample ID	Disease manifestation	Age/Sex	ZIKV IgM (serum)	CHIKV IgM and IgG (serum)	DENV IgM and IgG (serum)	Sample collection date	CHIKV qRT-PCR Ct value	Genome coverage depth	Ratio of *de novo* and African->Asian SNPs >
PCR reaction (H2O) C-						_	_	1641.92	_
Serum C-						_	_	1033.73	_
#199P195	Classical CHIKV	59/male	IgM ind	IgM- IgG+	IgM- IgG+	07-04-2016	31	7232.20	15/23
#97P95	Classical CHIKV	25/female	IgM -	IgM- IgG-	IgM- IgG+	06-01-2016	13	10538.00	9/8
#43P41	Classical CHIKV	71/male	IgM -	IgM- IgG-	IgM- IgG+	05-17-2016	13	6943.42	5/6
#89P87	Acute Tranv. Myelitis	62/male	IgM -	IgM+ IgG+	IgM- IgG+	05-31-2016	32	7653.14	8/59
#290P274	Optic Neuritis	43/male	IgM -	IgM- IgG-	IgM ind IgG-	07-25-2016	32	5749.48	0/20
#53P41	Acute Tranv. Myelitis	22/male	IgM -	IgM- IgG-	IgM- IgG+	03-30-2016	32	8298.55	8/133
#7P7	Polymyositis	22/male	IgM -	IgM+ IgG+	IgM- IgG-	03-30-2016	32	2201.24	2/7
#315P290	Polymyositis	30/male	IgM -	IgM+ IgG+	IgG- IgM-	08-16-2016	31	2701.50	4/39

### Polymorphisms and coinfection

**Fig 1:**
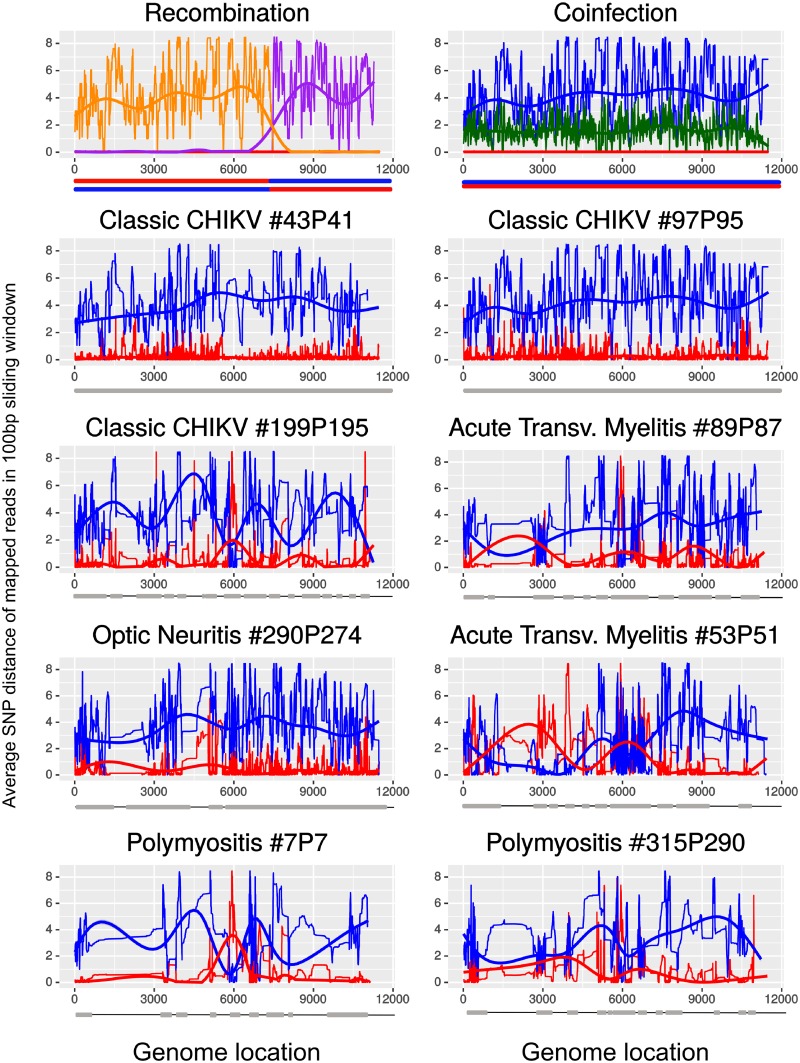
Dissimilarity plot of reads in 100bp windows against a reference ECSA (KP164568) and a Asian/Caribbean (KP164571.1) genome. Two upper plots were generated with simulated reads datasets in which we built a possible recombination (purple a genome with ECSA 5’ region and Asian/Caribbean 3’ region, orange a genome with Asian/Caribbean 5’ region and ECSA 3’ region) and coinfection (green—mixed reads from previous simulations in equal proportion representing coinfection and red signal from reads simulated from KP164568 ECSA genotype genome, blue—signal from reads simulated from KP164571.1 Asian/Caribbean genome) signal between ECSA and Asian/Caribbean genomes. Following plots correspond to the reads obtained to our 8 samples mapped against ECSA (red line) and Asian/Caribbean (blue line) genomes. We also added a trend line following the same colors to highlight the major patterns. Below each plot there is a grey bar representing the genome regions that we have coverage depth higher than 200x and thicker black line are regions where the coverage depth was lower than 200x.

All sequenced samples yielded a majority of reads mapping on the ECSA genotype reference genome (Fig 1). However, we detected that six out of eight samples (#199P195, #89P87, #290P274, #53P51, #7P7, #315P290) had an unusual high genetic variability (Fig 1). While three presented a much narrow variability (#43P41 and #97P45) (Fig 1).

**Fig 2:**
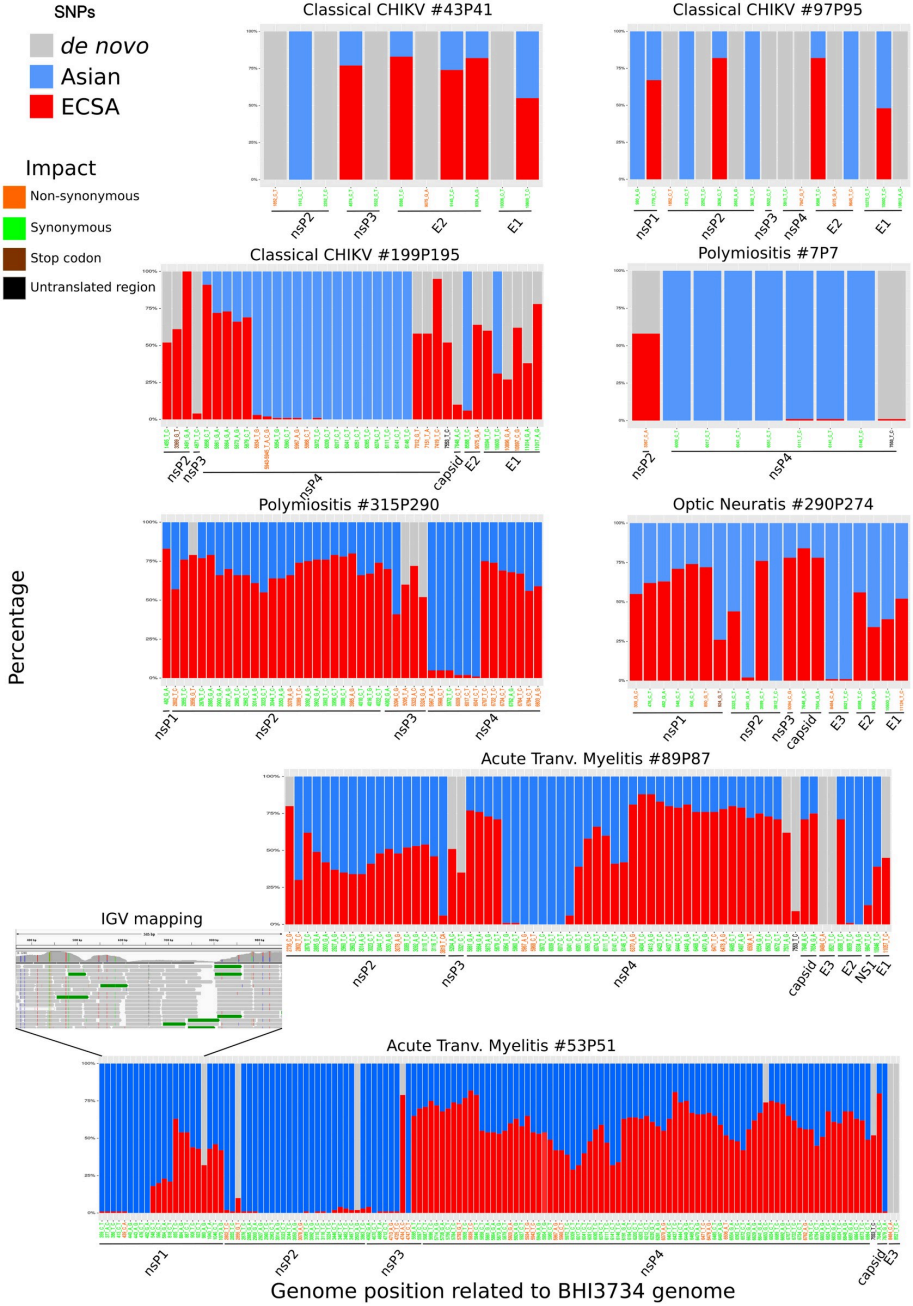
Single nucleotide polymorphisms in CHIKV genomes. All plots represent the SNPs and variants found having the K164568 ECSA genotype reference genome. Red bars represent the proportion of reads supporting the ECSA reference genome nucleotide, grey bars represent *de novo* SNPs (not present in either the ECSA and Asian/Caribbean reference genomes) and blue bars are SNPs found in the Asian/Caribbean reference genome. X-axis have the SNP position in relation to the ECSA reference CHIKV genome K164568 and their color are related with the impact of each SNP: orange are SNPs which generate a non-synonymous change; green are synonymous SNPs; brown are SNPs with generate a stop codon and black are SNPs in untranslated region. Black lines below x-axis denoted the SNP position relative to the mature CHIKV proteins.

To check whether viral genomic variability could be the result of new CHIKV *de novo* genomic variants or to any other source of variation (coinfection or recombination) we called and annotate SNPs using a ECSA lineage reference genome. Fig 2 shows the different genome regions in which highly supported nucleotide variability (coverage depth > 200x) was found, along with the amino acid variations of the different mature peptides. Most of the polymorphisms were concentrated in the nsP4 region. We found several SNPs inducing amino acid changes, compared to the ECSA mature peptides (N = 69, non-synonymous SNPs), but the majority of the SNPs are synonymous (N = 348). The samples 89P87 and 53P51 showed the largest number of SNPs (141) with several Asian/Caribbean variants, and regions with SNPs supporting either the ECSA or Asian/Caribbean genotypes. Most of the SNPs found correspond to the exact variants present in the Asian genotype reference genome. Four of these samples (with the exception of sample #7P7) presented at least 7 times more variants corresponding to the Asian/Caribbean genotype than *de novo* mutations (Table 1 and Fig 2). Thus, the high variability observed is likely derived from coinfection and/or recombination between the ECSA and Asian/Caribbean genotypes rather than from *de novo* mutations.

The inversion and average signal in the dissimilarity plot detected in several of the analysed samples may have been a result of new recombinant genomic variants between the ECSA and Asian/Caribbean genotypes or *bona fide* coinfection of these two genotypes (Fig 1 upper left panel). Our analysis revealed genomic regions covered by reads more similar to the Asian/Caribbean genome while other regions were more similar to the ECSA genome (Fig 1—89P87, 29074, 53P41, 7P7 and 315P290). We detected only very few reads containing both genotypes specific SNPs, at potential breaking points, varying from only 1 to 14 reads in samples 7P7 and 53P51 respectively (S2 Data). Additionally, we also found genomic regions that are mapped by roughly as many reads supporting both ECSA and Asian/Caribbean genotype in sample 89P87 (~2800-3200bp) and 53P51 (~5600-7300bp) (Fig 1). Thus, the vast majority of reads supporting uniquely the ECSA or the Asian/Caribbean genotype, with no or only few reads supporting both genotypes (single reads with ECSA and Asian/Caribbean genotype-specific SNPs) and the average signal found in the dissimilarity plot supports that coinfection, rather than recombination, has occurred.

### Phylogenetic relationships

**Fig 3:**
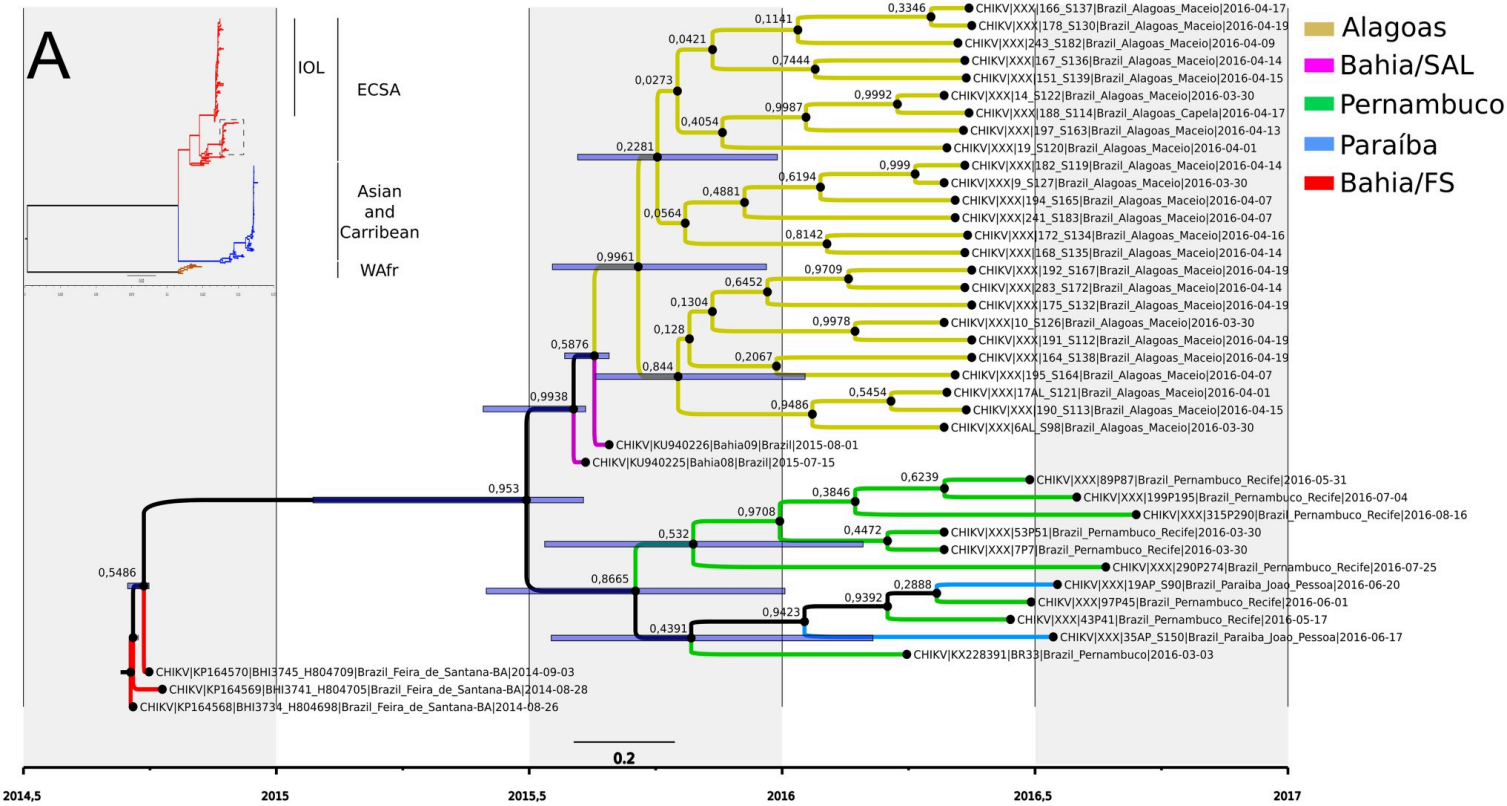
Phylogenetic reconstruction and dating of CHIKV strains circulating in Brazil. A) Genome-wide bayesian analysis with ECSA (red branches) and Asian/Caribbean (blue branches) lineages. Dotted square is the clade were the sampled obtained in this study clustered and the sequences selected for the molecular clock analysis. B) Specific bayesian reconstruction with relaxed molecular clock of ECSA genomes found in Brazil with the presumed index case from Feira de Santana—Bahia state.

The draft genomes recovered were clearly more represented by the African CHIKV genotype belonging to the ECSA lineage which entered Brazil at Feira de Santana—Bahia in 2014 (Fig 3A). Phylogenetic reconstruction showed that the genomes from Pernambuco state clustered with genomes from Paraíba, a north neighbouring state (Fig 3B). The two full genomes obtained from samples 43P41 and 97P45 clustered with high support with Paraíba state genomes while all other genomes clustered in a distinct clade (Fig 3B). Based on relaxed molecular clock estimates the common ancestor of Pernambuco samples dates between June 2015 and January 2016 with an average in September/October of 2015 (Fig 3B).

## Discussion

CHIKV infection has continually caused health and economic burden around the world and due to the continuous population flux this virus reached Brazil in 2014, spreading rapidly in a large naive human population [2,17,37]. Although some studies exist [16,19,38,39], only limited information is available on the epidemiology and evolution of CHIKV after its entrance in Brazil. In particular, we still do not know whether the two genotypes (ECSA and Asian/Caribbean) are spreading countrywide and what is their prevalence in different regions of the country. Furthermore, it is unclear whether these two genotypes cocirculate and whether coinfection occurs as has been reported for other arboviruses [40,41]. Here, through direct sequencing of CHIKV genomes from clinical samples, we found that the ECSA and the Asian/Caribbean genotypes are cocirculating, or at least have been cocirculating in the Northeast region of Brazil. Surprisingly, we also report, for the first time, data supporting simultaneous infection by two different genotypes in at least six clinical samples.

**Fig 4:**
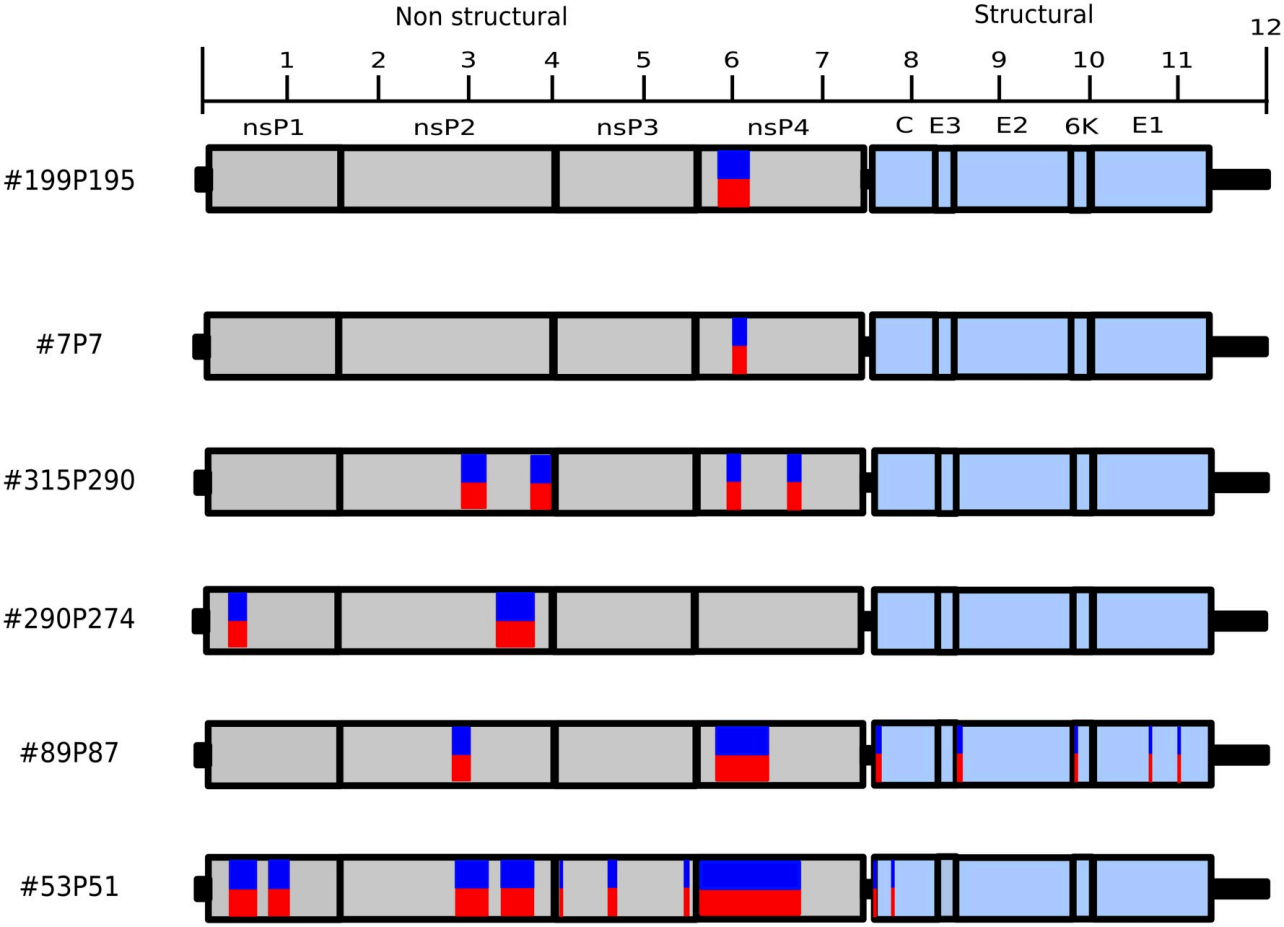
Schematic representation of the genomes sequenced showing the position where reads supported both ECSA and Asian/Caribbean CHIKV genotype. Red and blue boxes denotes regions with variants (ECSA and Asian/Caribbean respectively) as shown in Fig 2 but not showing the proportion of reads supporting each variant.

While, at first, both recombination and coinfection could be invoked to explain the presence of both ECSA and Asian/Caribbean SNPs in our samples, several lines of evidence support the coinfection hypothesis. The patchy distribution of genomic fragments sequenced from each genotype in the same sample could be taken as evidence of recombination (Figs 2 and 4). However, this pattern can also be obtained in the case of coinfection, assuming differences in the amplification dynamics coupled to dissimilar titers of each genotype (viremia) and the likely variation of viral genome integrity of each genotype. Thus, if these patterns were due to recombination, one would have to invoke multiple different recombination events, which we believe is highly unlikely. Finally, and perhaps more importantly, the extreme scarcity of reads supporting potential breakpoints (i.e. reads containing SNPs from both genotypes), together with samples whereby multiple genomic regions are mapped by similar proportions of reads from both genotypes (Fig 4) strongly suggest the coinfection hypothesis is the most likely explanation.

It has been shown that a host infected by different DENV viral genotypes can progress to a more severe disease [40]. CHIKV coinfection has so far been reported only with other viral species such as CHIKV and DENV, CHIKV and ZIKV or CHIKV, ZIKV and DENV [39,42–47] coinfected patients, but coinfection in humans generally occurs at a low rate [44]. Studies reporting coinfection by different DENV genotypes suggest a link between coinfection and more severe clinical symptoms. In one of these studies, three children diagnosed with meningitis were shown to be infected by two to three different DENV serotypes [41]. Another study showed that 40 patients out of 280 diagnosed with DENV were simultaneously coinfected by different serotypes, being associated with severe clinical manifestations [40]. Interestingly, the finding of *Ae*. *aegypti* mosquitoes coinfected by CHIKV and ZIKV can simultaneously transmit both viruses [47], supports the idea that during concomitant outbreaks coinfection may occurs [48]. Albeit our data clearly bring new insights into the molecular epidemiology of CHIKV it remains to be elucidated if coinfection by two different genotypes can have any consequence on viral fitness and disease progression and outcome.

In addition to coinfection likely driving the observed higher CHIKV variability, altered viral fitness resulting from point mutation-inducing structural changes may have also occurred. Thus we searched, in our draft genomes, for specific mutations known to exacerbate viral neurotropism [49,50]. We did not observe any potential amino acid changes in the CHIKV envelope (E2) glycoprotein, positions 67 to 82. Indeed, all obtained draft genomes, compared to publicly available sequences from ECSA and Asian/Carribean genotypes, were identical at this specific E2 glycoprotein region. Another CHIKV genomic region that may impact viral fitness and disease outcome is the RdRp, since specific changes can produce larger mutation clouds [51,52]. However, no known mutation that affects the RdRp fidelity was found in our samples. Therefore, there is no evidence that the RdRp mutations observed here had a major impact on the observed genetic variability.

Finally, molecular clock analysis of the ECSA genotype genomes estimated the entrance of the virus in the Pernambuco state between June/July 2015 and November/December 2015 (HPD 95%), in very close agreement with the firsts CHIKV infection cases in Pernambuco detected in June 2015 reaching its peak in August of the same year [19]. The agreement between molecular clock inferences and epidemiological data shows that the sequencing of viral genomes coupled with evolutionary studies brings a wealthy of data to understand the evolutionary dynamics of the virus and make inferences about its spread. Therefore, genomic epidemiology studies can provides several key information to guide public health efforts focused on epidemic surveillance and outbreak control [53].

Clearly, one limitation of this study relies on the small number of sequenced samples. Hence, the possible link between coinfection by multiple CHIKV genotypes and severe symptoms/disorders should be further investigated in a large sample set to statistically evaluate the relationship of coinfection and disease manifestation. Taken together, our findings highlight the importance of sequencing viral genomes directly from clinical samples using high throughput sequencing, as it allows a fine-grained assessment of viral variability present during infection.

## Supporting information

S1 Table Primers sets used for whole CHIKV genome amplification. (XLSX)

S2 Table SNPs called from each genome and associated statistics. (XLSX)

S1 Data R script used to generate dissimilarity plot shown in Fi 1. (R)

S2 Data Alignment of chimeric reads against two CHIKV reference genomes from ECSA and Asian/Caribbean genotypes. (ZIP)

S1 Fig Similarity plot of the six available CHIKV genomes aligned (three from ECSA and three from Asian/Caribbean genotypes). (PNG)

## References

[1] Rupp JC, Sokoloski KJ, Gebhart NN, Hardy RW. Alphavirus RNA synthesis and non-structural protein functions. J Gen Virol. 2015;96: 2483–2500. 10.1099/jgv.0.000249 26219641 PMC4635493

[2] Costa-da-Silva AL, Ioshino RS, Petersen V, Lima AF, Cunha MDP, Wiley MR, et al First report of naturally infected Aedes aegypti with chikungunya virus genotype ECSA in the Americas. PLoS Neglected Trop Dis. 2017;11: e0005630. 10.1371/journal.pntd.0005630 PMC5470658 28614394

[3] Chevillon C, Briant L, Renaud F, Devaux C. The Chikungunya threat: an ecological and evolutionary perspective. Trends Microbiol. 2008;16: 80–88. 10.1016/j.tim.2007.12.003 18191569

[4] Lebrun G, Chadda K, Reboux A-H, Martinet O, Gaüzère B-A. Guillain-Barré syndrome after chikungunya infection. Emerg Infect Dis. 2009;15: 495–496. 10.3201/eid1503.071482 19239775 PMC2681104

[5] Weaver SC, Lecuit M. Chikungunya virus and the global spread of a mosquito-borne disease. New Engl J Med. 2015;372: 1231–1239. 10.1056/NEJMra1406035 25806915

[6] Agarwal A, Vibha D, Srivastava AK, Shukla G, Prasad K. Guillain-Barre syndrome complicating chikungunya virus infection. J Neurovirology. 2017;23: 504–507. 28194661 10.1007/s13365-017-0516-1

[7] Brizzi K. Neurologic Manifestation of Chikungunya Virus. Curr Infect Dis Reports. 2017;19: 6. 10.1007/s11908-017-0561-1 28233188

[8] Pinheiro TJ, Guimarães LF, Silva MTT, Soares CN. Neurological manifestations of Chikungunya and Zika infections. Arq de neuro-psiquiatria. 2016;74: 937–943. 10.1590/0004-282X20160138 27901259

[9] Crosby L, Perreau C, Madeux B, Cossic J, Armand C, Herrmann-Storke C, et al Severe manifestations of chikungunya virus in critically ill patients during the 2013–2014 Caribbean outbreak. Int J Infect Dis: IJID : Off Publ Int Soc Infect Dis. 2016;48: 78–80. 10.1016/j.ijid.2016.05.010 27208636

[10] Simon F, Javelle E, Cabie A, Bouquillard E, Troisgros O, Gentile G, et al French guidelines for the management of chikungunya (acute and persistent presentations). November 2014. Med et Mal Infect. 2015;45: 243–263. 10.1016/j.medmal.2015.05.007 26119684

[11] ROSS RW. The Newala epidemic. III. The virus: isolation, pathogenic properties and relationship to the epidemic. J Hyg. 1956;54: 177–191. 13346078 10.1017/s0022172400044442 PMC2218030

[12] Chen R, Puri V, Fedorova N, Lin D, Hari KL, Jain R, et al Comprehensive Genome Scale Phylogenetic Study Provides New Insights on the Global Expansion of Chikungunya Virus. J Virol. 2016;90: 10600–10611. 10.1128/JVI.01166-16 27654297 PMC5110187

[13] Weaver SC. Arrival of chikungunya virus in the new world: prospects for spread and impact on public health. PLoS Neglected Trop Dis. 2014;8: e2921. 10.1371/journal.pntd.0002921 PMC4072586 24967777

[14] Morrison TE. Reemergence of chikungunya virus. J Virol. 2014;88: 11644–11647. 10.1128/JVI.01432-14 25078691 PMC4178719

[15] Nunes MRT, Faria NR, de Vasconcelos JM, Golding N, Kraemer MUG, de Oliveira LF, et al Emergence and potential for spread of Chikungunya virus in Brazil. BMC Med. 2015;13: 102 10.1186/s12916-015-0348-x 25976325 PMC4433093

[16] Rodrigues Faria N, Lourenço J, Marques de Cerqueira E, Maia de Lima M, Pybus O, Carlos Junior Alcantara L. Epidemiology of Chikungunya Virus in Bahia, Brazil, 2014–2015. PLoS Curr. 2016;8. 10.1371/currents.outbreaks.c97507e3e48efb946401755d468c28b2 PMC4747681 27330849

[17] Charlys da Costa A, Thézé J, Komninakis SCV, Sanz-Duro RL, Felinto MRL, Moura LCC, et al Spread of Chikungunya Virus East/Central/South African Genotype in Northeast Brazil. Emerg Infect Dis. 2017;23. 10.3201/eid2310.170307 PMC5621546 28930031

[18] Cunha MDP, Santos CAD, de Neto DFL, Schanoski AS, Pour SZ, Passos SD, et al Outbreak of chikungunya virus in a vulnerable population of Sergipe, Brazil-A molecular and serological survey. J Clin Virol: Off Publ Pan Am Soc Clin Virol. 2017;97: 44–49. 10.1016/j.jcv.2017.10.015 PMC7617034 29100064

[19] Magalhaes T, Braga C, Cordeiro MT, Oliveira ALS, Castanha PMS, Maciel APR, et al Zika virus displacement by a chikungunya outbreak in Recife, Brazil. PLoS Neglected Trop Dis. 2017;11: e0006055. 10.1371/journal.pntd.0006055 PMC5697888 29108009

[20] Cunha MS, Cruz NVG, Schnellrath LC, Medaglia MLG, Casotto ME, Albano RM, et al Autochthonous Transmission of East/Central/South African Genotype Chikungunya Virus, Brazil. Emerg Infect Dis. 2017;23: 1737–1739. 10.3201/eid2310.161855 28930027 PMC5621531

[21] White SK, Mavian C, Salemi M, Morris JG, Elbadry MA, Okech BA, et al A new “American” subgroup of African-lineage Chikungunya virus detected in and isolated from mosquitoes collected in Haiti, 2016. PloS one. 2018;13: e0196857 10.1371/journal.pone.0196857 29746539 PMC5944945

[22] Lanciotti RS, Kosoy OL, Laven JJ, Panella AJ, Velez JO, Lambert AJ, et al Chikungunya virus in US travelers returning from India, 2006. Emerg Infect Dis. 2007;13: 764–767. 10.3201/eid1305.070015 17553261 PMC2738459

[23] Lanciotti RS, Kosoy OL, Laven JJ, Velez JO, Lambert AJ, Johnson AJ, et al Genetic and serologic properties of Zika virus associated with an epidemic, Yap State, Micronesia, 2007. Emerg Infect Dis. 2008;14: 1232–1239. 10.3201/eid1408.080287 18680646 PMC2600394

[24] Edwards T, Del Carmen Castillo Signor L, Williams C, Larcher C, Espinel M, Theaker J, et al Analytical and clinical performance of a Chikungunya qRT-PCR for Central and South America. Diagn Microbiol Infect Dis. 2017;89: 35–39. 10.1016/j.diagmicrobio.2017.06.001 28633900 PMC5560405

[25] Bolger AM, Lohse M, Usadel B. Trimmomatic: a flexible trimmer for Illumina sequence data. Bioinforma. 2014;30: 2114–2120. 10.1093/bioinformatics/btu170 PMC4103590 24695404

[26] Martin DP, Murrell B, Golden M, Khoosal A, Muhire B. RDP4: Detection and analysis of recombination patterns in virus genomes. Virus Evol. 2015;1: vev003 10.1093/ve/vev003 27774277 PMC5014473

[27] Lole KS, Bollinger RC, Paranjape RS, Gadkari D, Kulkarni SS, Novak NG, et al Full-Length Human Immunodeficiency Virus Type 1 Genomes from Subtype C-Infected Seroconverters in India, with Evidence of Intersubtype Recombination. J Virol. 1999;73: 152–160. 9847317 10.1128/jvi.73.1.152-160.1999 PMC103818

[28] Iles JC, Njouom R, Foupouapouognigni Y, Bonsall D, Bowden R, Trebes A, et al Characterization of Hepatitis C Virus Recombination in Cameroon by Use of Nonspecific Next-Generation Sequencing. J Clin Microbiol. 2015;53: 3155–3164. 10.1128/JCM.00483-15 26202126 PMC4572555

[29] Langmead B, Salzberg SL. Fast gapped-read alignment with Bowtie 2. Nat methods. 2012;9: 357–359. 10.1038/nmeth.1923 22388286 PMC3322381

[30] Wickham H. ggplot2: Elegant Graphics for Data Analysis (Use R). 2nd Printing Springer; 2009.

[31] Danecek P, Auton A, Abecasis G, Albers CA, Banks E, DePristo MA, et al The variant call format and VCFtools. Bioinformatics. 2011;27. 10.1093/bioinformatics/btr330 PMC3137218 21653522

[32] Cingolani P, Platts A, Wang LL, Coon M, Nguyen T, Wang L, et al A program for annotating and predicting the effects of single nucleotide polymorphisms, SnpEff: SNPs in the genome of Drosophila melanogaster strain w1118; iso-2; iso-3. Fly. Taylor & Francis; 2012;6: 80–92. 10.4161/fly.19695 PMC3679285 22728672

[33] Huang W, Li L, Myers JR, Marth GT. ART: a next-generation sequencing read simulator. Bioinforma. 2011;28: 593–4. 10.1093/bioinformatics/btr708 PMC3278762 22199392

[34] Bouckaert R, Heled J, Kühnert D, Vaughan T, Wu C-H, Xie D, et al BEAST 2: a software platform for Bayesian evolutionary analysis. PLoS Comput Biol. 2014;10: e1003537 10.1371/journal.pcbi.1003537 24722319 PMC3985171

[35] Darriba D, Taboada GL, Doallo R, Posada D. jModelTest 2: more models, new heuristics and parallel computing. Nat methods. 2012;9: 772. 10.1038/nmeth.2109 PMC4594756 22847109

[36] Metsky HC, Matranga CB, Wohl S, Schaffner SF, Freije CA, Winnicki SM, et al Zika virus evolution and spread in the Americas. Nature. 2017;546: 411–415. 10.1038/nature22402 28538734 PMC5563848

[37] Vega-Rúa A, Lourenço-de-Oliveira R, Mousson L, Vazeille M, Fuchs S, Yébakima A, et al Chikungunya virus transmission potential by local Aedes mosquitoes in the Americas and Europe. PLoS Neglected Trop Dis. 2015;9: e0003780. 10.1371/journal.pntd.0003780 PMC4439146 25993633

[38] Dias JP, Costa M da CN, Campos GS, Paixão ES, Natividade MS, Barreto FR, et al Seroprevalence of Chikungunya Virus after Its Emergence in Brazil. Emerg Infect Dis. 2018;24. 10.3201/eid2404.171370 PMC5875253 29553317

[39] Pessôa R, Patriota JV, de Lourdes de Souza M, Felix AC, Mamede N, Sanabani SS. Investigation Into an Outbreak of Dengue-like Illness in Pernambuco, Brazil, Revealed a Cocirculation of Zika, Chikungunya, and Dengue Virus Type 1. Medicine. 2016;95: e3201 10.1097/MD.0000000000003201 27015222 PMC4998417

[40] Dhanoa A, Hassan SS, Ngim CF, Lau CF, Chan TS, Adnan NAA, et al Impact of dengue virus (DENV) co-infection on clinical manifestations, disease severity and laboratory parameters. BMC Infect Dis. 2016;16: 406 10.1186/s12879-016-1731-8 27514512 PMC4982428

[41] Marinho PES, Bretas de Oliveira D, Candiani TMS, Crispim APC, Alvarenga PPM, Castro FC dos S, et al Meningitis Associated with Simultaneous Infection by Multiple Dengue Virus Serotypes in Children, Brazil. Emerg Infect Dis. 2017;23. 10.3201/eid2301.160817 PMC5176234 27983492

[42] Rodriguez-Morales AJ, Villamil-Gómez WE, Franco-Paredes C. The arboviral burden of disease caused by co-circulation and co-infection of dengue, chikungunya and Zika in the Americas. Travel Med Infect Dis. 2016;14: 177–179. 10.1016/j.tmaid.2016.05.004 27224471

[43] Villamil-Gómez WE, Rodríguez-Morales AJ, Uribe-García AM, González-Arismendy E, Castellanos JE, Calvo EP, et al Zika, dengue, and chikungunya co-infection in a pregnant woman from Colombia. Int J Infect Dis : IJID : Off Publ Int Soc Infect Dis. 2016;51: 135–138. 10.1016/j.ijid.2016.07.017 27497951

[44] Carrillo-Hernández MY, Ruiz-Saenz J, Villamizar LJ, Gómez-Rangel SY, Martínez-Gutierrez M. Co-circulation and simultaneous co-infection of dengue, chikungunya, and zika viruses in patients with febrile syndrome at the Colombian-Venezuelan border. BMC Infect Dis. 2018;18: 61 10.1186/s12879-018-2976-1 29382300 PMC5791178

[45] Furuya-Kanamori L, Liang S, Milinovich G, Soares Magalhaes RJ, Clements ACA, Hu W, et al Co-distribution and co-infection of chikungunya and dengue viruses. BMC Infect Dis. 2016;16: 84 10.1186/s12879-016-1417-2 26936191 PMC4776349

[46] Deeba F, Afreen N, Islam A, Naqvi IH, Broor S, Ahmed A, et al. Co-infection with Dengue and Chikungunya Viruses. Current Topics in Chikungunya. InTech; 2016.

[47] Brito CAA, Azevedo F, Cordeiro MT, Marques ETA, Franca RFO. Central and peripheral nervous system involvement caused by Zika and chikungunya coinfection. PLoS Neglected Trop Dis. 2017;11: e0005583. 10.1371/journal.pntd.0005583 PMC5509110 28704365

[48] Göertz GP, Vogels CBF, Geertsema C, Koenraadt CJM, Pijlman GP. Mosquito co-infection with Zika and chikungunya virus allows simultaneous transmission without affecting vector competence of Aedes aegypti. PLoS Neglected Trop Dis. 2017;11: e0005654. 10.1371/journal.pntd.0005654 PMC5469501 28570693

[49] Gardner CL, Ebel GD, Ryman KD, Klimstra WB. Heparan sulfate binding by natural eastern equine encephalitis viruses promotes neurovirulence. Proc Natl Acad Sci United States Am. 2011;108: 16026–16031. 10.1073/pnas.1110617108 PMC3179095 21896745

[50] Gardner CL, Hritz J, Sun C, Vanlandingham DL, Song TY, Ghedin E, et al Deliberate attenuation of chikungunya virus by adaptation to heparan sulfate-dependent infectivity: a model for rational arboviral vaccine design. PLoS Neglected Trop Dis. 2014;8: e2719. 10.1371/journal.pntd.0002719 PMC3930508 24587470

[51] Coffey LL, Vignuzzi M. Host alternation of chikungunya virus increases fitness while restricting population diversity and adaptability to novel selective pressures. J Virol. 2011;85: 1025–1035. 10.1128/JVI.01918-10 21047966 PMC3020036

[52] Xiao Y, Dolan PT, Goldstein EF, Li M, Farkov M, Brodsky L, et al Poliovirus intrahost evolution is required to overcome tissue-specific innate immune responses. Nat Commun. 2017;8: 375 10.1038/s41467-017-00354-5 28851882 PMC5575128

[53] Pybus OG, Rambaut A. Evolutionary analysis of the dynamics of viral infectious disease. Nat Rev Genet. 2009;10: 540–550. 10.1038/nrg2583 19564871 PMC7097015

