# Supplementary figures and images for: Genome sequencing reveals coinfection by multiple chikungunya virus genotypes in a recent outbreak in Brazil
Source: PLoS Negl Trop Dis. 2019 May 16;13(5):e0007332. doi: 10.1371/journal.pntd.0007332 (PMC6541278; doi:10.1371/journal.pntd.0007332)

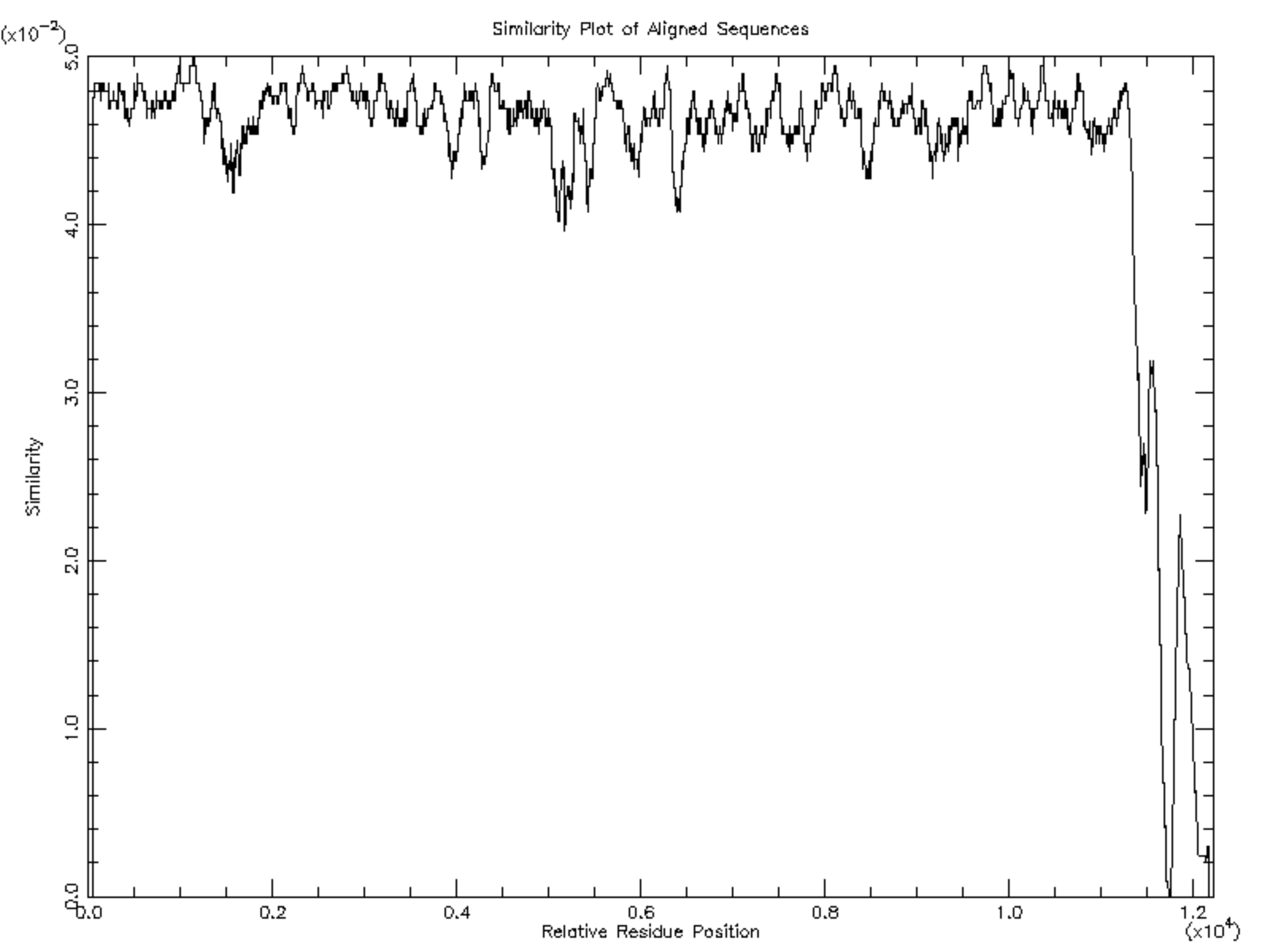

Supplement: S1 Fig — (PNG) [file pntd.0007332.s005.png]
